# Quality of Life After Orthognathic Surgery in Patients with Cleft: An Overview of Available Patient-Reported Outcome Measures

**DOI:** 10.1177/10556656211067120

**Published:** 2021-12-17

**Authors:** Roan L. M. Ploumen, Samuel H. Willemse, Ronald E. G. Jonkman, Jitske W. Nolte, Alfred G. Becking

**Affiliations:** 1 Department of Oral & Maxillofacial Surgery, 26066 Amsterdam UMC, University of Amsterdam, Amsterdam, The Netherlands; 2 Department of Orthodontics, 1192 Academic Centre for Dentistry Amsterdam (ACTA), Amsterdam, The Netherlands

**Keywords:** quality of life, orthognathic surgery, craniofacial morphology

## Abstract

**Objective:**

Measuring the impact of orthognathic surgery on quality of life is of significant importance in patients with cleft deformities. Standardized tools such as patient-reported outcome measures (PROMs) are needed to fully comprehend patients’ needs and perceptions. Therefore, the availability of reliable, valid, and comprehensive questionnaires for patients is essential. The aim of this study is to identify PROMs measuring the impact of orthognathic surgery on quality of life in patients with cleft deformities and to evaluate the identified PROMs.

**Methods:**

A systematic search of the literature was performed according to the preferred reporting items for systematic reviews and meta-analysis guidelines. All validated PROMs, regarding the impact of orthognathic surgery on quality of life in patients with cleft deformities, were identified and assessed according to the quality criteria proposed for measurement properties of health status questionnaires.

**Results:**

An electronic search yielded 577 articles. After a full-text review of 87 articles, 4 articles met the inclusion criteria, comprising 58 PROMs. Of these 58 PROMs, 1 PROM (the CLEFT-Q) has been validated to measure the impact of orthognathic surgery on patients with a facial cleft. Evaluation of methodological quality of the included articles and assessment of the measurement properties of the CLEFT-Q show that the CLEFT-Q scores relatively good for all available measurement properties, making it suitable for immediate use.

**Conclusion:**

The CLEFT-Q was found to be the only valid instrument so far to measure the impact of orthognathic surgery on the quality of life in patients with cleft deformities.

## Introduction

Congenital anomalies occur in 2% to 3% of all newborns. Cleft lip (alveolus) and/or palate and isolated cleft palate (CP) defects are the most common variants of craniofacial anomalies, occurring in between every 1 of 700 till 1 in 1000 births (Mossey and Modell, 2012; Mai et al., 2019).

Patients with orofacial clefts may be hampered by feeding difficulties and deviant facial appearance. Speech, facial growth, and tooth eruption disturbances may occur. Treatment needs staging and lasts from birth to adulthood. It is complex and includes a multidisciplinary approach (Hussein et al., 2012). Despite optimal treatment, maxillary hypoplasia occurs in ∼25% (reported range 14%-50%) of cleft patients as a result of intrinsic deformity, facial growth patterns, genetic inheritance, and scar tissue (Chigurupati, 2012). Orthognathic surgery can be a useful intervention for restoring function and aesthetic appearance (Krey et al., 2013).

The goal of treatment in patients is to reduce the specific impairment and to achieve an increase in oral function and psychological and social well-being, resulting in an increased general quality of life (QOL). It is reported that orthognathic surgery can increase the QOL in general patient groups with dentofacial deformities (Soh and Narayanan, 2013). However, research on the impact of orthognathic surgery on QOL in patients with a cleft is scarce. An increase in QOL results from the fulfillment of a patient's treatment need (Sinko et al., 2005).

To understand and evaluate patients’ perceptions and treatment needs, a reliable and valid measurement tool is essential. A standardized tool to measure the impact of treatment on QOL is the patient-reported outcome measure (PROM), which includes measurements on generic health or disease-specific aspects (Black, 2013). To evaluate surgically relevant outcomes in patients with a cleft, specifically developed and validated PROMs are required (Cano et al., 2004).

Previous systematic searches have been performed to find valid PROMs for orthognathic treatment or for patients with cleft deformities (Eckstein et al., 2011; Klassen et al., 2012; Zamboni et al., 2019). No search has yet been conducted to find a PROM measuring the specific impact of orthognathic surgery on QOL in patients with cleft lip and palate.

This systematic search has 2 objectives. The first objective is to identify valid PROMs for measuring the impact of orthognathic surgery on the QOL in patients. The second objective is to evaluate and assess the quality of these PROMs.

## Methods

This review was performed based on the preferred reporting items for systematic reviews and meta-analysis (PRISMA)-statement (www.prisma-statement.org) (Moher et al., 2010).

### Search

The comprehensive search was performed by a clinical librarian working in the affiliated medical library. The comprehensive search was performed in the databases PubMed/MEDLINE, EMBASE (Ovid), Cochrane Library, and Web of Science from the inception of the databases until 11 January 2021. The terms used were: orthognathic surgery, orthognathic surgical procedures, maxillofacial abnormalities, dento-maxillary orthopedics AND cleft lip, CP, cleft alveolus, cleft lip alveolus and palate, congenital deformity, dentofacial deformity AND PRO, surveys and questionnaires, PRO measure, PROM, QOL, life quality, health-related QOL (HRQOL), and psychometrics. The complete search strategies for all online databases can be found in Appendix 1. Limits were placed on the search to exclude non-English citations and articles.

### Eligibility Criteria

All studies were evaluated by title, keywords, and abstract by 2 reviewers (RP and SW). Discords were resolved by consensus. Full-text reviews of the selected articles were independently performed by 2 reviewers (RP and SW). Discords were again resolved by consensus. Articles were selected for full review according to the following a priori eligibility criteria.

Inclusion criteria:

- Studies evaluating PROM.
- Studies with descriptions about the evaluation or construct of the measurement tool.
- Studies with extensive descriptions of questionnaires used in orthognathic surgery or in patients with dentofacial deformities, or patients with a cleft, lip (alveolus), and/or palate.
- Studies with patient sample sizes were described.

Exclusion criteria:

- Questionnaires evaluating family conditions only.

### Identification and Assessment of Validation of PROMs

Inclusion criteria*:*

- Instruments measuring QOL validated for patients with cleft deformities and for the impact of orthognathic surgery on QOL.

Exclusion criteria*:*

- Instruments under construction.
- Instruments using parents’ or caregivers proxy measures instead of patient-reported data.
- Ad hoc instruments.
- Instruments are not available in English language.

After identification of instruments measuring QOL, a follow-up search was performed by 2 authors RP and SW to check if instruments were both validated for patients with cleft deformities and for the impact of orthognathic surgery on QOL. Unvalidated instruments were excluded.

### Quality Assessment

The methodological quality of the studies, describing the PROMs suitable for quality assessment, were evaluated with the consensus-based standards for the selection of health status measurement instruments (COSMIN) risk of bias checklist (Mokkink et al., 2018; Terwee et al., 2018; Prinsen et al., 2018). The COSMIN checklist contains 10 boxes to assess the included studies’ methodological quality of the measurement properties, using a 4-point scale ranging from very good, adequate, doubtful till inadequate. Measurement properties not possible to evaluate were rated not applicable. A detailed description of use, evaluation, and scores of the COSMIN risk of bias checklist can be found in the COSMIN methodology for systematic reviews of patient-reported outcome measures (PROMs) user manual (Mokkink et al., 2018; Prinsen et al., 2018; Terwee et al., 2018).

PROMs measuring the impact of orthognathic surgery on QOL in patients with cleft deformities were assessed using the quality criteria proposed for measurement properties of health status questionnaires (Terwee et al., 2007). Positive, intermediate, negative or no information available ratings can be given to the measurement properties: (1) content validity, (2) internal consistency, (3) criterion validity, (4) construct validity, (5) reproducibility, (6) responsiveness, (7) floor and ceiling effects, and (8) interpretability. The description of the defined quality criteria and rating system is detailed described (Terwee et al., 2007).

## Results

**Figure 1.**
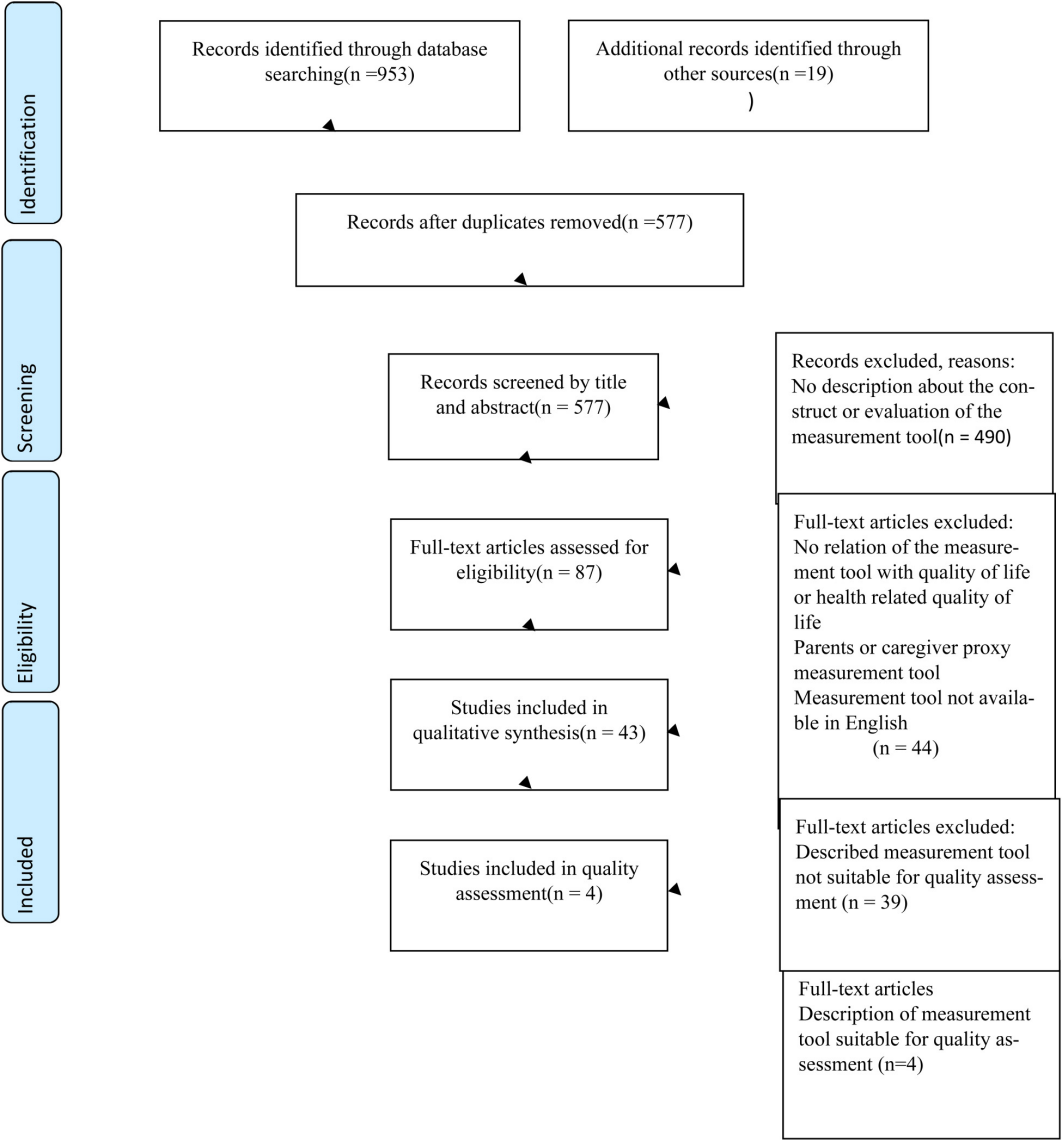
Preferred reporting items for systematic reviews and meta-analysis (PRISMA) 2009 flow diagram.

After removing duplicate studies, 577 articles were independently screened by title and abstract by 2 reviewers. A total of 87 articles were eligible for full-text review. Forty-three articles met the inclusion criteria, describing the construction or validation of PROMs used in orthognathic or cleft studies. Of these 43 articles, 4 articles were found suitable for quality assessment. The corresponding PRISMA flow diagram is presented in Figure 1.

**Figure 2.**
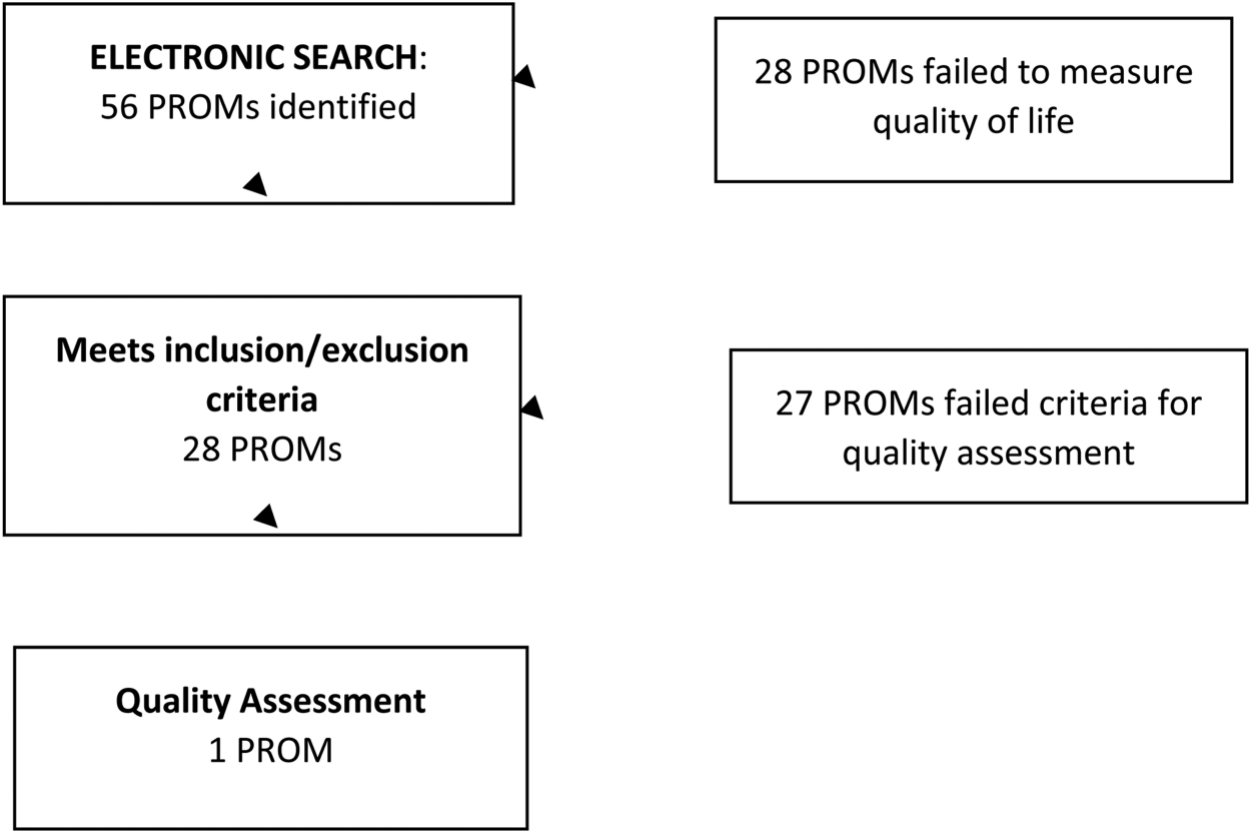
Flow diagram of patient-reported outcome measures (PROMs).

After the screening and reviewing of 577 articles, 56 PROMs were identified. Whereas 28 PROMs measured QOL, the other 28 PROMs were parent proxy instruments or not available in English or measured different dimensions or health issues, for example, social avoidance and distress. Of these 28 PROMs measuring QOL, 13 measured QOL only, 2 were validated for measuring the impact of orthognathic treatment on QOL, 13 were validated for measuring QOL in patients with a cleft, and only 1 PROM, the CLEFT-Q, was validated for measuring the impact of orthognathic treatment on QOL in patients with a cleft (Table 1 and Figure 2).

**Table 1.** Identified Instruments Measuring Quality of Life.

#	Questionnaire	Orthognathic treatment	Patients with cleft deformities
1	OQLQ	+	−
2	FACE-Q	+	−
3	OIDP	−	−
	OHIP		
4	−14	−	−
5	−49	−	−
6	SF-36	−	−
7	VAS	−	+
8	WHOQOL-BREF	−	−
9	CLEFT-Q	+	+
	COHIP		
10	−11	−	−
11	−38	−	−
12	-SF 19	−	+
13	SWLS	−	−
	CPQ		
14	8–11	−	+
15	11–14	−	+
16	POQL	−	−
17	VELO	−	+
18	PEDSQL	−	+
19	PROMIS	−	+
20	QLACA	−	+
21	PVRQOL	−	+
	YQOL		
22	FD	−	+
23	CS	−	+
24	KINDL-R	−	−
25	PIDAQ	−	−
26	EUROQOL EQ-5D	−	−
27	MOHRQOL	−	+
28	CHASQ	−	+

Abbreviations: OQLQ, orthognathic quality of life questionnaire; OIDP, oral impact on daily performance; OHIP-14/49, oral health impact profile 14/49; SF-36, short-form health survey-36; VAS, visual analog scale; WHOQOL-BREF, World Health Organization quality of life-BREF; COHIP 11/38/SF19, child oral health impact profile 11/38/short-form health survey-19; SWLS, satisfaction with life scale; CPQ 8-11/11-14, child perception questionnaire 8-11/11-14; POQL, pediatric oral health-related quality of life; VELO, VPI effects on life outcomes; PEDSQL, pediatric quality of life inventory; PROMIS, patient-reported outcomes measurement information system; QLACA, quality of life in adolescent with cleft assessment; PVRQOL, pediatric voice-related quality of life; YQOL FD/CS, youth quality of life instrument facial differences/craniofacial surgery; PIDAQ, psychosocial impact of dental aesthetics questionnaire; EUROQOL EQ-5D, EURO quality of life 5 dimensions; MOHRQOL, Michigan oral health-related quality of life; CHASQ, cleft hearing, appearance and speech questionnaire.

### Risk of Bias Checklist

The CLEFT-Q has been identified as a validated PROM measuring the impact of orthognathic surgery on QOL in a patient with a cleft lip (alveolus) and/or palate and is described in 4 studies: Wong Riff et al. (2017), Klassen et al. (2018), and Harrison et al. (2019). It consists of 12 different scales measuring appearance (of the nose, teeth, lips, jaws, cleft lip scar, and the face), speech function (social, school, speech distress, psychological), and HRQOL. Lower scale scores are associated with facial appearance dissatisfaction, speech problems, and a need for future treatment regarding cleft-related problems (Wong Riff et al., 2017; Klassen et al., 2018). Three studies, Klassen et al. (2018), and Harrison et al. (2019), extensively describe the construct and validation of the CLEFT-Q.

Results of the COSMIN risk of bias checklist of the 4 included studies show that the methodological quality of the studies scores is “very good.” Wong Riff et al. (2017) described only the study protocol of the development of the CLEFT-Q (Wong Riff et al., 2017) therefore no scores were given for this study (Table 2).

**Table 2.** Consensus-Based Standards for the Selection of Health Status Measurement Instruments (COSMIN) Checklist Methodological Quality of Included Studies.

	PROM development	Content validity	Structural validity	Internal consistency	Cross-cultural validity	Reliability	Measurement error	Criterion validity	Hypothesis testing for construct validity	Responsiveness
CLEFT-Q										
Wong 2017	-	-	-	-	-	-	-	-	-	-
Tsangaris 2017	Very good	Very good	-	-	-	-	-	-	-	-
Klassen 2018	-	-	Very good	Very good	-	Very good	-	-	Very good	-
Harrison 2019	-	-	-	-	-	-	-	-	Very good	-

Rating: -, nonapplicable.

### Quality Assessment of PROMs

Only 1 PROM, the CLEFT-Q, was assessed with the quality criteria proposed for measurement properties of health status questionnaires (Terwee et al., 2007). The quality criteria for the 8 attributes of instrument properties: (1) content validity, (2) internal consistency, (3) criterion validity, (4) construct validity, (5) reproducibility, (6) responsiveness, (7) floor and ceiling effects, and (8) interpretability were evaluated and are summarized in Table 3 (Terwee et al., 2012).

**Table 3.** Summary of the Assessment of the Measurement Properties of PROM Measuring Impact of Orthognathic Surgery in QOL in Patients with a Cleft.

Questionnaire	(1) Content validity	(2) Internal consistency	(3) Criterion validity	(4) Construct validity	(5) Reproducibility		(6) Responsiveness	(7) Floor or ceiling effect	(8) Interpretability
					(a) Agreement	(b) Reliability			
CLEFT-Q	+	+	?	+	?	+	?	?	0

Rating: +, positive; 0, intermediate; -, poor; ?, no information available.

PROM, patient-reported outcome measures; QOL, quality of life.

Specification:

1. The content validity is described extensively. Target population, concepts, and item selection are discussed, therefore content validity is considered positive (Klassen et al., 2018).
2. The internal consistency shows Cronbach α values ranging from .89 to .96 (Cronbach's alpha is considered good between .70 and .95). Regarding factor analyses, the sample size of the CLEFT-Q was >7 times the number of items, and the sample size was >100 (Klassen et al., 2018). Therefore, internal consistency is considered positive.
3. Currently, there is no information available to evaluate the criterion validity of the CLEFT-Q. In the study protocol published by the developers, the criterion validity is planned to be further elaborated in phase 3 of the construction of the CLEFT-Q (Wong Riff et al., 2017).
4. Construct validity of the CLEFT-Q is assessed by a priori hypotheses. Rasch analysis provided evidence of reliability and validity for 12 of 13 scales (Klassen et al., 2018). Construct validity of the CLEFT-Q is considered positive because specific hypotheses were formulated and at least 75% of the results are in accordance with these hypotheses.
5. Reproducibility can be divided into (5a) agreement and (5b) reliability. No information was found for the agreement. The reliability Person separation index values were ≥0.85 for 10/12 scales (intraclass correlation coefficient [ICC] or ICC or weighted Kappa >0.70 is considered good) (Klassen et al., 2018). The reliability of the CLEFT-Q is therefore considered positive.
6. The responsiveness is also planned to be further elaborated in phase 3 of the construction of the CLEFT-Q (Wong Riff et al., 2017; Harrison et al., 2019). At the moment there is no information available for this attribute.
7. In all 4 articles, floor or ceiling effects are not mentioned, therefore assessment is not applicable for floor or ceiling effects.
8. For the last attribute, the interpretability, the mean and standard deviation (SD) scores are defined in 4 relevant subgroups of patients (Harrison et al., 2019). The definition of the minimal important change (MIC) is scheduled in phase 3 of the CLEFT-Q construction (Wong Riff et al., 2017). With the criteria “mean and SD scores presented off at least four relevant subgroups of patients and no MIC defined” (Terwee et al., 2007), the interpretability of the CLEFT-Q is assessed as “intermediate.”

## Discussion

The purpose of this systematic search was to identify valid PROMs for measuring the impact of orthognathic surgery on QOL in patients with cleft deformities and to evaluate the quality of the valid PROMs. A systematic search of the literature was conducted and resulted in 577 articles of which 4 articles met the inclusion criteria describing the construct and validation of the only valid PROM found: the CLEFT-Q. The quality assessment showed 4 good results out of 8 criteria, with 4 criteria yet under construction, making the CLEFT-Q the only valid PROM available for clinical use at this moment.

### Results Compared to Previous Research

In previously conducted searches for instruments measuring the QOL in patients with a cleft in general, 5 and 6 validated instruments were found by Eckstein and Klassen, respectively (Eckstein et al., 2011; Klassen et al., 2012). Since these searches have been performed until 2011, more cleft-specific PROMs have subsequently been constructed over time, resulting in the CLEFT-Q and 13 other validated instruments (visual analog scale [VAS], child oral health impact profile short-form health survey-19 [COHIP-SF19], child perception questionnaire 8-11 [CPQ 8-11], Child Perception Questionnaire 11-14 [CPQ 11-14], VPI effects on life outcomes [VELO], pediatric QOL inventory [PEDSQL], patient-reported outcomes measurement information system [PROMIS], QOL in adolescent with cleft assessment [QLACA], pediatric voice-related QOL [PVRQOL], youth QOL instrument facial differences [YQOL FD], youth QOL instrument craniofacial surgery [YQOL CS], MOHRQOL, and the cleft hearing, appearance and speech questionnaire [CHASQ]; Table 1). In addition, several cleft-parents or caregivers-report instruments and cleft instruments under construction were identified in the present search, but did not meet the inclusion criteria of this study. In a previously conducted search by Zamboni et al. (2019), regarding instruments measuring the impact of orthognathic surgery on QOL 7 PROMs were found. Most of those questionnaires were not properly validated, and only the orthognathic QOL questionnaire (OQLQ) met the inclusion criteria of the present study.

PROMs such as the oral health impact profile 14 (OHIP-14), short-form health survey-36 (SF-36), World Health Organization QOL-BREF (WHOQOL-BREF), sence of coherence questionnaire-29, VAS, and patient atisfaction questionnaire have been used in single or multiple studies investigating the impact of orthognathic surgery on QOL (Zamboni et al., 2019). These PROMs have not been validated for measuring orthognathic QOL in patients with a cleft.

### CLEFT-Q

The only valid PROM found in this search, the CLEFT-Q, scores mainly “very good” after evaluating the methodological quality with the COSMIN checklist (Terwee et al., 2012). Quality assessment of the CLEFT-Q demonstrates a positive score in content validity, internal consistency, construct validity, and reliability and an intermediate score in interpretability. There is no information yet available for criterion validity, agreement, responsiveness, and floor- and ceiling effects. The proposed quality criteria by Terwee et al. (2007), consider content validity as 1 of the most important measurement properties. They state that when the content validity has shown to be adequate, the questionnaire is allowed to be used. The CLEFT-Q scores positive in content validity and several other criteria and is therefore considered as immediately usable for clinicians (Klassen et al., 2018).

The CLEFT-Q has been translated into multiple languages and scores very high in cross-cultural validity (Tsangaris et al., 2018). Non-English instruments were outside the design of this study, and thus not included in the assessment.

### Limitations of This Study

The majority of cleft-orientated studies or constructed cleft-specific PROMs focus on patients aged 0 to 21 years. Due to a large number of young patients, several PROMs make use of the reports of parents or caregivers proxy (Klassen et al., 2012). It appears that there is a good concordance between the reports of parents or caregivers proxy and children in relationship to children's oral health (Wilson-Genderson et al., 2007; Clawson et al., 2013). With an average age of 17 years or more during the orthognathic surgical intervention (Yamaguchi et al., 2016), parents or caregivers proxy focused PROMs are considered not contributive.

Non-English articles and instruments were excluded in the present study, therefore possible PROMs could have been overlooked. The researchers conducted follow-up searches for every PROM identified as thoroughly as possible and previous systematic reviews have been scanned for PROMs, but cannot completely rule out an incomplete literature search.

No specific orthognathic procedures have been determined for inclusion and exclusion a priori. All articles describing skeletal corrections of the jaws and face have been included. In the article describing further construct validation of the CLEFT-Q, only “Jaw surgery” has been described regarding orthognathic surgery (Harrison et al., 2019). It is a suggestion for future studies to specifically describe the orthognathic procedure executed in patients.

## Conclusion

The CLEFT-Q was found to be the only valid instrument to measure the impact of orthognathic surgery on the QOL in patients with cleft deformities. Further development of the CLEFT-Q is needed to be able to assess all the measurement properties with respect to orthognathic surgery.

## Supplemental Material

sj-docx-1-cpc-10.1177_10556656211067120 - Supplemental material for Quality of Life After Orthognathic Surgery in Patients with Cleft: An Overview of Available Patient-Reported Outcome Measures Click here for additional data file. Supplemental material, sj-docx-1-cpc-10.1177_10556656211067120 for Quality of Life After Orthognathic Surgery in Patients with Cleft: An Overview of Available Patient-Reported Outcome Measures by Roan L. M. Ploumen, Samuel H. Willemse, Ronald E. G. Jonkman, Jitske W. Nolte and Alfred G. Becking in The Cleft Palate Craniofacial Journal
