# Supplementary material for: Quality of Life After Orthognathic Surgery in Patients with Cleft: An Overview of Available Patient-Reported Outcome Measures
Source: Cleft Palate Craniofac J. 2021 Dec 17;60(4):405–12. doi: 10.1177/10556656211067120 (PMC10018051; doi:10.1177/10556656211067120)
Supplement: sj-docx-1-cpc-10.1177_10556656211067120 - Supplemental material for Quality of Life After Orthognathic Surgery in Patients with Cleft: An Overview of Available Patient-Reported Outcome Measures [file sj-docx-1-cpc-10.1177_10556656211067120.docx]

**APPENDIX 1**

PubMed 275 hits:

("Orthognathic Surgery"[Mesh] OR "Orthognathic Surgical Procedures"[Mesh] OR "Orthodontics"[Mesh] OR "Jaw/surgery"[Mesh] OR "Maxillofacial Abnormalities"[Mesh] OR orthognath*[tiab] OR jaw surger*[tiab] OR corrective surger*[tiab] OR surg*[tiab] OR dento alveolar orthopedic*[tiab] OR dento facial orthopedic*[tiab] OR dentoalveolar orthopedic*[tiab] OR dentofacial orthopedic*[tiab] OR dentomaxillary orthopedic*[tiab] OR orthodont*[tiab])

AND

("Cleft Lip"[Mesh] OR "Cleft Palate"[Mesh] OR cleft*[tiab] OR congenital deformit*[tiab] OR dentofacial deformit*[tiab])

AND

("Patient Reported Outcome Measures"[Mesh] OR "Surveys and Questionnaires"[Mesh] OR PRO measure*[tiab] OR questionnaire*[tiab] OR measur*[tiab] OR Patient Reported Outcome*[tiab] OR PROM[tiab])

AND

("Quality of Life"[Mesh] OR quality of life[tiab] OR life quality[tiab] OR health-related quality of life[tiab] OR hrqol[tiab] OR qol[tiab] OR psychometrics[tiab])  **"2021/01/12"[Date - Publication])**

**EMBASE (Ovid):** update

Database(s): **Embase Classic+Embase**1947 to 2021 January 11
Search Strategy:

| **#** | **Searches** | **Results** |
| --- | --- | --- |
| 1 | exp orthognathic surgery/ or orthodontics/ or exp orthodontic procedure/ or jaw/su or exp face malformation/ or (orthognath* or jaw surger* or corrective surger* or surg* or dento alveolar orthopedic* or dento facial orthopedic* or dentoalveolar orthopedic* or dentofacial orthopedic* or dentomaxillary orthopedic* or orthodont*).ti,ab,kw. | 3041561 |
| 2 | exp cleft lip/ or cleft palate/ or exp cleft lip palate/ or (cleft* or congenital deformit* or dentofacial deformit*).ti,ab,kw. | 69032 |
| 3 | patient-reported outcome/ or exp questionnaire/ or (PRO measure* or questionnaire* or measur* or patient reported outcome* or PROM).ti,ab,kw. | 5527094 |
| 4 | exp "quality of life"/ or (quality of life or life quality or health-related quality of life or hrqol or qol or psychometrics).ti,ab,kw. | 640428 |
| 5 | 1 and 2 and 3 and 4 | 367 |
| 6 | limit 5 to conference abstract status | 62 |
| 7 | 5 not 6 | 305 |

Cochrane Library

Cochrane Database of Systematic Reviews

Issue 1 of 12, January 2021

1hit

Cochrane Central Register of Controlled Trials

Issue 1 of 12, January 2021

ID Search Hits

#1 (orthognathic surg* OR maxillofacial abnormalit* OR orthognath* OR jaw surger* OR corrective surger* OR surg* OR dento alveolar orthopedic* OR dento facial orthopedic* OR dentoalveolar orthopedic* OR dentofacial orthopedic* OR dentomaxillary orthopedic* OR orthodont*):ti,ab,kw 254371

#2 (cleft* or congenital deformit* or dentofacial deformit*):ti,ab,kw 1369

#3 (PRO measure* or questionnaire* or measur* or patient reported outcome* or PROM):ti,ab,kw 540999

#4 (quality of life or life quality or health-related quality of life or hrqol or qol or psychometric*):ti,ab,kw 132434

#5 #1 and #2 and #3 and #4 25

Web of Science

Timespan: All years. Indexes: SCI-EXPANDED, SSCI, A&HCI, ESCI.

421 hits:

TOPIC: (orthognathic surg* OR maxillofacial abnormalit* OR orthognath* OR jaw surger* OR corrective surger* OR surg* OR dento alveolar orthopedic* OR dento facial orthopedic* OR dentoalveolar orthopedic* OR dentofacial orthopedic* OR dentomaxillary orthopedic* OR orthodont*)

AND

TOPIC: (cleft* or congenital deformit* or dentofacial deformit*)

AND

TOPIC: (PRO measure* or questionnaire* or measur* or patient reported outcome* or PROM)

AND

TOPIC: (quality of life or life quality or health-related quality of life or hrqol or qol or psychometric*)
